# Novel aspects of grapevine response to phytoplasma infection investigated by a proteomic and phospho-proteomic approach with data integration into functional networks

**DOI:** 10.1186/1471-2164-14-38

**Published:** 2013-01-17

**Authors:** Paolo Margaria, Simona Abbà, Sabrina Palmano

**Affiliations:** 1 Istituto di Virologia Vegetale, CNR, Strada delle Cacce 73, 10135, Torino, Italy

**Keywords:** *Vitis vinifera*, Flavescence dorée, Recovery, 2-DE

## Abstract

**Background:**

Translational and post-translational protein modifications play a key role in the response of plants to pathogen infection. Among the latter, phosphorylation is critical in modulating protein structure, localization and interaction with other partners. In this work, we used a multiplex staining approach with 2D gels to study quantitative changes in the proteome and phosphoproteome of Flavescence dorée-affected and recovered ‘Barbera’ grapevines, compared to healthy plants.

**Results:**

We identified 48 proteins that differentially changed in abundance, phosphorylation, or both in response to Flavescence dorée phytoplasma infection. Most of them did not show any significant difference in recovered plants, which, by contrast, were characterized by changes in abundance, phosphorylation, or both for 17 proteins not detected in infected plants. Some enzymes involved in the antioxidant response that were up-regulated in infected plants, such as isocitrate dehydrogenase and glutathione S-transferase, returned to healthy-state levels in recovered plants. Others belonging to the same functional category were even down-regulated in recovered plants (oxidoreductase GLYR1 and ascorbate peroxidase). Our proteomic approach thus agreed with previously published biochemical and RT-qPCR data which reported down-regulation of scavenging enzymes and accumulation of H_2_O_2_ in recovered plants, possibly suggesting a role for this molecule in remission from infection. Fifteen differentially phosphorylated proteins (| ratio | > 2, p < 0.05) were identified in infected compared to healthy plants, including proteins involved in photosynthesis, response to stress and the antioxidant system. Many were not differentially phosphorylated in recovered compared to healthy plants, pointing to their specific role in responding to infection, followed by a return to a steady-state phosphorylation level after remission of symptoms. Gene ontology (GO) enrichment and statistical analysis showed that the general main category “response to stimulus” was over-represented in both infected and recovered plants but, in the latter, the specific child category “response to biotic stimulus” was no longer found, suggesting a return to steady-state levels for those proteins specifically required for defence against pathogens.

**Conclusions:**

Proteomic data were integrated into biological networks and their interactions were represented through a hypothetical model, showing the effects of protein modulation on primary metabolic ways and related secondary pathways. By following a multiplex-staining approach, we obtained new data on grapevine proteome pathways that specifically change at the phosphorylation level during phytoplasma infection and following recovery, focusing for the first time on phosphoproteome changes during pathogen infection in this host.

## Background

Flavescence dorée (FD) is an economically important grapevine disease in Europe caused by phytoplasmas, plant phloem-limited pathogens in the class Mollicutes [1]. Flavescence dorée is transmitted by *Scaphoideus titanus* (Ball) and its potential to cause epidemic outbreaks makes the associated phytoplasma (FDp) a quarantine pathogen in Europe. All *Vitis vinifera* cultivars are susceptible to FD phytoplasma (FDp). Among the typical symptoms, infected plants show yellowing, downward rolling of the leaves, stunting and lack of lignifications of the new shoots at the end of the vegetative season. Flower withering and premature berry shrivelling can cause heavy yield losses and reduced wine quality. However, the severity and incidence of symptoms associated with FDp infection can vary according to the type of cultivar. ‘Barbera’ is a typical Italian cultivar, mainly cultivated in Piedmont (northern Italy), but also in important wine-producing areas outside Italy [2] producing economically important high quality wines. ‘Barbera’ shows high sensitivity to FDp infection: besides showing severe symptoms at the beginning of the vegetative season (early syndrome), infected plants clearly show typical symptoms of the disease in summer, which can frequently result in whole plants turning purple. Interestingly, as well as this high incidence, ‘Barbera’ shows a high recovery phenotype (spontaneous remission of symptoms) after initial infection with FDp [3].

Over recent years, genomic and proteomic strategies have been successfully used to analyse plant-pathogen interactions. In particular, proteomic approaches have largely evolved in pursuit of the functional assignment of proteins expressed during phytopathogenic interactions [4]. Despite the great economic importance of phytoplasma diseases, proteomics has only recently been applied to this class of aetiological agents, in mulberry [5,6], grapevine [7] and Mexican lime tree [8]. For grapevine-phytoplasma interactions, the only proteomic analysis so far published studied the response of the cv. Nebbiolo to FDp infection. ‘Nebbiolo’ is generally considered as being more tolerant to FDp infection, showing milder symptoms than ‘Barbera’. In this work we used a more sensitive staining approach than the one we used previously [7] which allowed a better characterization of both the proteome and phosphoproteome of a susceptible cultivar. We also extended the analysis to recovered plants, with the aim of highlighting new putative proteins involved in susceptibility/tolerance to phytoplasma infection.

Studies on post-translational modifications (PTMs) are considered important in clarifying pathogen/host interactions, and their role in the dynamic adaptation of plants to different conditions has been demonstrated [9]. Among them, reversible protein phosphorylation of serine, threonine and tyrosine residues is the most common protein modification, and has a crucial role in regulating the plant response toward different abiotic and biotic stresses [10]. Transcriptomic approaches recently identified several protein kinases and a tyrosine phosphatase which were up-regulated in grapevines infected by Bois noir phytoplasma [11,12]. In addition, a serine/threonine protein kinase transcript was up-regulated in Mexican lime tree infected by *Candidatus* Phytoplasma aurantifolia [13], and a tyrosine phosphate protein was up-regulated in *Morus alba* infected by phytoplasmas [5]. These data suggest an important role for phosphorylation events in the response to phytoplasma infection. However, to the best of our knowledge, no data are yet available on the kinase/phosphatase targets and on PTMs of grapevine proteins in response to pathogens, and specifically to phytoplasma infection and recovery. Recovery is an interesting phenomenon that consists in the remission of disease symptoms from a previous infection, which has also been reported in phytoplasma-infected plants [14]. In grapevine, this phenomenon is accompanied by the inability to detect the phytoplasma in the recovered plant tissues. So far, very limited data are available on the possible molecular mechanisms involved. A role for H_2_O_2_, which accumulates in tissues of recovered plants following down-regulation of catalase and ascorbate peroxidase scavenging enzymes, is supported by biochemical and RT-qPCR data [15]. The study of recovery is now of major interest, in view of its potential use as a control strategy against grapevine phytoplasmas, due to the absence of genetic resources for resistance.

In this work, we monitored changes in the total proteome and phosphoproteome of FDp-healthy, infected and recovered grapevines through different staining techniques. Spots showing qualitative and quantitative changes were trypsin-digested and the resulting peptides analysed by Matrix-Assisted Laser Desorption Ionization-Time Of Flight (MALDI-TOF-TOF). The differentially expressed proteins were integrated into functional networks in order to represent their biological significance in the host-pathogen interaction. Our data gave new valuable insights into specific protein modulations that occur in grapevine during progression of Flavescence dorée and after recovery from the disease, providing valuable proteomic data on this poorly characterized phenomenon.

## Results and discussion

**Figure 1:**
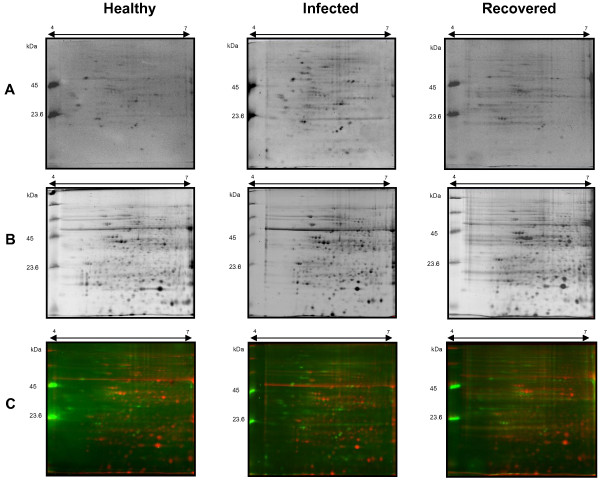
**2-DE proteomic maps of proteins and phosphoproteins of *Vitis vinifera *leaves from healthy, infected and recovered plants.** Protein extracts were separated on 7 cm long IPG strips with a pH gradient from 4 to 7, followed by SDS-PAGE on 12% polyacrilamide gels. Panel **A**: ProQ Diamond staining for phosphorylated protein detection. Panel **B**: Sypro^®^ Ruby staining for total protein detection. Panel **C**: overlap of the two images (in red Sypro^®^ Ruby, in green ProQ Diamond).

Grapevine midrib tissues from healthy, infected and recovered plants were considered for the extraction of total proteins. In order to minimize the environmental and sample variability we collected the plants in the same vineyard area, nearby each other and in the same day. Grapevines were scored for symptoms and screened for phytoplasma and virus infection by molecular assays, as described in Methods section. In all the samples, we excluded the presence of seven out of the eight viruses for which we tested, with the exception of Grapevine fleck virus (GFkV), which was present in each vine in the vineyard. Furthermore, its presence was not expected to interfere with our investigation, as GFkV is generally latent and asymptomatic in grapevines [16]. Nine 2-DE gels (three biological X three technical replications) for each condition (healthy, infected and recovered plants) were run, stained with two dyes and analysed. The ProQ Diamond stain (Invitrogen, Carlsbad, CA, USA), which is specific for phosphorylated serine, threonine and tyrosine protein residues, was used to identify phosphorylated proteins and to study their response to phytoplasma infection and in recovered plants. Once the first stain was removed, total proteins were detected on the same gels using the fluorescent Sypro^®^ Ruby stain (Invitrogen). Using PDQuest gel analysis software, up to 130 phospho-spots and 450 total-spots were detected in all the gels, using the two dyes respectively. Phospho-proteins were mainly concentrated in the 40–60 kDa range and had a p*I* mostly between 5-6 (Figure 1A) due to acidification of the proteins, as previously reported in phospho-proteomic studies [17,18]. Sypro^®^ Ruby stained proteins were evenly distributed on the gels, covering almost all the p*I* and molecular weight (MW) range (Figure 1B). Using a multiplex staining approach on the same gel, we were able to overlap the images and precisely match them together, in order to normalize the data and distinguish a lightly phosphorylated, high abundance protein from a heavily phosphorylated, low abundance protein (Figure 1C). From the same Figure 1, we could also check the specificity of the dyes; for example, several high-abundance spots detected by Sypro Ruby were not stained by ProQ Diamond.

**Figure 2:**
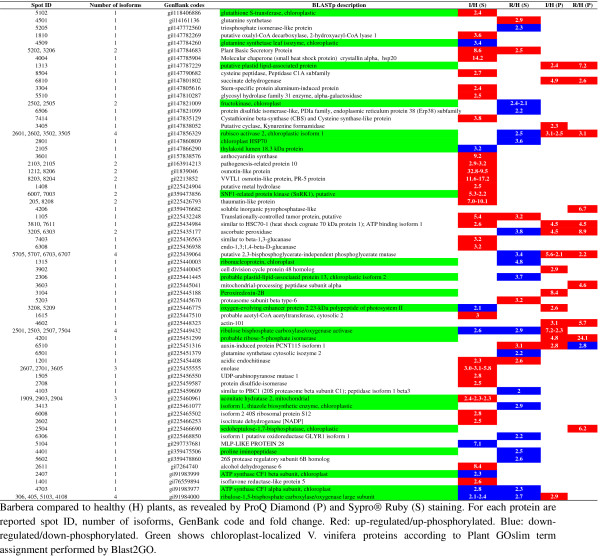
**Differentially expressed/phosphorylated proteins in phytoplasma-infected (I) and recovered (R) grapevines in comparison to healthy (H) ones. **Differentially expressed proteins (S) have been revealed by Sypro^®^ Ruby stain and differentially phosphorylated proteins (P) have been revealed by ProQ Diamond stain. For each protein spot ID, number of isoforms, GenBank code and fold change are reported. Red: up-regulated/up-phosphorylated. Blue: down-regulated/down-phosphorylated. Green shows chloroplast-localized *V. vinifera* proteins according to Plant GOslim term assignment performed by Blast2GO.

For spot identification, the 2-DE electrophoresis was coupled with MALDI TOF/TOF analysis. This system is suitable for the identification of the most abundant proteins in the spot analysed. However, this technical limit did not hamper the ability to provide a description of the main changes that occur in the grapevine proteome and phosphoproteome during phytoplasma infection and recovery (Figure 2, Additional file 1).

**Figure 3:**
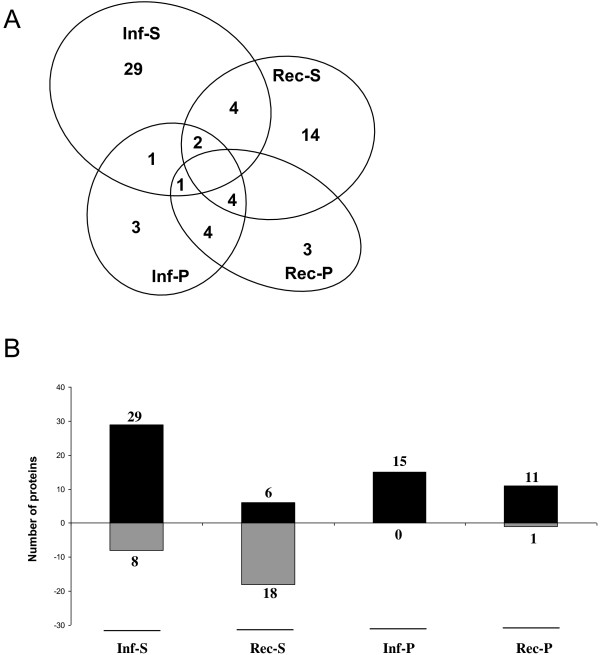
**Protein distribution and expression trend in infected and recovered plants. A**) Venn diagram showing the distribution of the differentially expressed proteins in each category. **B**) Induced and repressed grapevine proteins in response to infection and in recovered plants. Inf: infected. Rec: recovered. S: Sypro^®^ Ruby staining (total proteins). P: ProQ Diamond staining (phosphorylated proteins).

In Figure 3A, the Venn diagram shows the distribution of all the differentially regulated proteins found in the infected and recovered category, pointing out the presence of specific proteins characterizing each single status, as a well as the shared ones. In the discussion about differential regulation of the proteins, we used “up- or down-regulated” for the changes in abundance and “up- or down-phosphorylated” for the changes in the phosphorylation state.

### Proteome and phosphoproteome changes in FDp-infected grapevines

#### Proteome changes

A previous study analysed the response of the ‘Nebbiolo’ grapevine proteome to phytoplasma infection using a Coomassie Blue staining approach [7]. In this work we used the more sensitive Sypro^®^ Ruby stain, which improved analysis and detection of differentially expressed proteins. The nature of the spots was successfully determined by mass spectrometry analysis: some of them matched to the same protein, indicating the presence of multiple isoforms (Figure 2).

**Figure 4:**
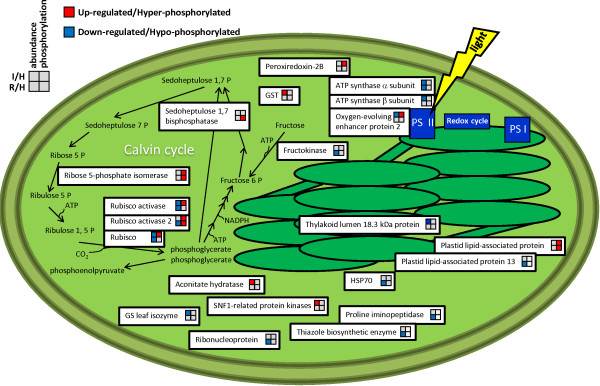
**Grapevine proteins with predicted plastid localization changing their quantitative and/or phosphorylation levels in infected and recovered plants.** The model gives an overview of the quantitative and qualitative (phosphorylation) changes in chloroplast-localized proteins, showing their involvement in specific metabolic pathways and predicted integration into biological networks according to the KEGG database. The expression trends are represented using a coloured legend in the upper left corner of the figure.

Twenty-nine out of the 37 differentially expressed proteins identified in FDp-infected samples were up-regulated, whereas 8 showed a down-regulation with respect to healthy plants (Figure 3B). Due to the high number of proteins, we focus below on the most significant members of each category, and discuss them according to previous reports in the literature. Many of the identified proteins were involved in metabolism and energy processes, confirming that these pathways are highly stressed by phytoplasmas [7]. In particular, several proteins involved in photosynthesis were down-regulated: two related to the dark reactions, ribulose bisphosphate carboxylase/oxygenase activase (gi|225449432) and ribulose-1,5-bisphosphate carboxylase/oxygenase (Rubisco, gi|91984000), and three related to the light-dependent reactions of photosystem II (PS II), chloroplastic adenosine-5'-triphosphate (ATP) synthase CF1 alpha subunit, (gi|91983977), ATP synthase CF1 beta subunit and oxygen-evolving enhancer protein 2 (OEE2p). Another case of co-expression of the ATP synthase alpha subunit and OEE2p has recently been reported in apoplectic and esca proper-affected grapevine plants [19]. Phytoplasma infection is well known to cause marked inhibition of photosynthetic activities in grapevine [11,12]. In particular, it has been demonstrated that RubisCO, the key enzyme of the Calvin-Benson cycle, is down-regulated at the transcriptional level [11] and also in its catalytic activity [20] in Bois noir phytoplasma-infected grapevine. Our data clearly showed the major changes affecting photosynthesis and, consequently, chloroplasts of infected leaves (Figure 4), further supporting the theory that chloroplasts may be key players in symptom development [21].

Multiple isoforms of enolase and aconitase enzymes were found to be differentially expressed in infected leaves. The enolase protein has been found to be responsive to several biotic [22] and abiotic stresses [23,24] in different plant species. Up-regulation of enolase might also increase the production of energy, which is needed in response to phytoplasma infection, as suggested in other systems [23]. Aconitase (spots 1909/2903/2904) catalyzes the reversible isomerization of citrate to isocitrate. This protein was found to be involved in response to abiotic stress [24,25] and in the regulation of carbon flow between the citric acid cycle and synthetic sucrose pathways [26]. The influence of aconitase on the citrate acid cycle probably has effects on the related glyoxylate cycle. In fact, we found the up-regulation of an oxalyl-coA decarboxylase, which is involved in glyoxylate metabolism and participates in the degradation of oxalic acid, a by-product accumulated under stress conditions [27].

Spot 2602 was identified as being isocitrate dehydrogenase. This protein shares 89% identity with the homologous protein in *Arabidopsis thaliana* (locus NP_176768), for which a fundamental role in redox signalling and the regulation of pathogen responses has been recently described [28]. A growing body of evidence suggests that redox homeostasis is a metabolic interface between stress perception and physiological responses [29]. Our results suggest a critical role for the cytosolic NADP(H) status in the colonized grapevine cells as a tool by which phytoplasma-triggered changes in the cellular redox state activate defence responses.

Several pathogenesis related proteins belonging to class PR-5 (thaumatins, osmotins), PR-10 and PR-17 (plant basic secretory protein) were up-regulated in infected tissues. PR-5 proteins were previously reported in grapevines infected by FDp [7], as well as by fungal pathogens [19,30,31], and upon abiotic stress [19]. Among the other stress responsive proteins, we also found Heat shock protein 70 (Hsp70). This protein is fundamental in developmental processes and response to abiotic stresses; moreover, various studies have reported that Hsp70 expression in plants is induced upon infection with pathogens [32].

Spot 3304 corresponded to an aluminium-induced protein. The same protein was found to be up-regulated in the grapevine cv. Razegui under salt stress [33]. Interestingly, our data also suggest a role for this protein in grapevine-phytoplasma biotic interactions. No data are so far available in the literature on its specific role inside the plant cell. A translationally controlled tumour protein (spot 1105) was also strongly up-regulated. This protein may participate together with SNF1-related protein kinase (SnRK1) (spot 6007, 7003) in the regulation of gene expression in response to stress [34].

Consistent with the importance of plant cell wall modifications in response to pathogen invasion, the up-regulation of proteins involved in cell wall re-organization was present in FDp-infected grapevines. An alpha endo-1,3;1,4-beta-D-glucanase (spot 6308), a beta 1,3-glucanase (spot 7403) and two glycosyl hydrolases (spots 5510 and 1201) were up-regulated. Our data are consistent with the results of previous mRNA analyses of grapevine cultivars [11,12,35] and periwinkle [36] infected by phytoplasmas. Alterations in plant cell walls and the localization of several compounds such as callose, suberin, lignin and polyphenols have been reported in woody hosts, such as plum and apple plants, during phytoplasma infection [37]. Our data likely suggest a role for these compounds and, in general, for cell wall modifications in grapevine-phytoplasma interactions and possibly in defence-related processes.

Among the responsive proteins identified in infected ‘Barbera’ plants, eight proteins have already been reported in FDp-infected ‘Nebbiolo’ [7]: thaumatin, osmotin, plant basic secretory protein, cysteine synthase, peptidase, Rubisco activase, dienelactone hydrolase, and ATPase β subunit. The protein expression trends were also in accordance between the two cultivars. The differential expression of these proteins may reflect a common role in the phytoplasma-grapevine interaction in both cultivars.

#### Phosphoproteome changes

Statistical analysis identified 15 differentially-phosphorylated proteins in infected vs. healthy plants (Figure 2). All of them showed an up-phosphorylation trend (Figure 3B). Several differentially phosphorylated spots corresponded to proteins involved in the photosynthetic apparatus. Multiple isoforms of RubisCO activase (Figure 2) were highly phosphorylated. A similar pattern was described in *Arabidopsis* following wounding stress [38]. In contrast, RubisCO activases were reported to be down-phosphorylated following treatment with chitosan, a compound commonly used as an elicitor mimicking fungal biotic stress, whereas no differences were observed after treatment with benzothiadiazole (BTH) [38]. The role of the phosphorylated isoforms is not known at present, although in some cases it may relate to a functional regulation of enzyme activity or turn-over. The molecular target of Rubisco activase, the Rubisco carboxylase/oxigenase large subunit (spot 5103), was also up-phosphorylated. Phosphorylation of Rubisco subunits has been demonstrated to be critical for activity, association and assembly of the Rubisco multicomponent complex [39,40]. In agreement with our results, the Rubisco large chain has previously been found to be phosphorylated in rice leaves in response to heat stress [18]. Some other proteins involved in light-chain reactions and energy production were phosphorylated (Figure 4), suggesting a role for phosphorylation in the regulation of several proteins involved in these processes. We can speculate that phosphorylation may function as a signal for protein degradation, leading to the inhibition of the photosynthetic apparatus and finally to the repression of these pathways in infected leaves.

Alteration of the light-chain reaction is strictly related to alterations in electron transfer and generation of reactive oxygen species [41]. Phosphorylation of antioxidant enzymes, including ascorbate peroxidase (spots 3205, 6303) and peroxiredoxin (spot 3104) proteins, showed that phosphorylation is involved in antioxidant defence in grapevine leaves. In agreement with our observations, up-phosphorylation of several proteins, including ascorbate peroxidase, was reported in *Arabidopsis* following induction of oxidative stress [38]. Increased activity for ascorbate peroxidases, after phosphorylation by specific kinases, was also reported in water-stressed maize leaves [42].

Peroxiredoxins (Prx) are a family of peroxidases found in all organisms and represent central elements of the antioxidant defence system [43]. The peroxiredoxin-5 proteins have been widely studied in mammalian cells and have been implicated in a broad range of functions, such as protection of DNA against damage by oxidants, inhibition of apoptosis and transduction in signalling pathways [44]. Phosphorylation of peroxiredoxin proteins was found in rice leaves in response to abiotic stress [18]. Our data suggests a role in protection from oxidative stress in phytoplasma-infected plants.

A PAP-fibrillin protein was previously found to be up-regulated in grapevine tissues infected by phytoplasmas [7]. Here we found differential phosphorylation of a putative plastid lipid-associated protein belonging to the PAP-fibrillin superfamily (spot 1313) in infected tissues, suggesting a role of both forms in response to phytoplasmas. Differential phosphorylation of PAP-fibrillin proteins has previously been reported in Arabidopsis in response to bacterial infection [45] and following treatment with the BTH elicitor [38].

Among the proteins involved in response to stress, an HSC70-1 protein was up-phosphorylated. These proteins are involved in protein targeting and degradation [46]. Remarkably, an HSC70 protein was strongly phosphorylated in *Arabidopsis* following treatment with mimickers of biotic stress [38], and a role in multiple plant environmental responses has been described [47]. Our data seem to confirm that HSC70-1 may function as a molecular switch for the response against bacterial challenge. Very recently, HSC70-1 was found to be necessary in establishing basal expression levels of several ABA-responsive genes, suggesting that this chaperone might also be involved in ABA signalling events [48].

### Proteome and phosphoproteome changes in recovered plants

#### Proteome changes

Analysis of the whole proteome of recovered plants showed differential expression for 24 proteins when compared to healthy grapevines. Six were common to the 37 proteins found in the comparison between healthy and infected plants. Among them, the plant basic secretory protein (gi|147784683), translationally-controlled tumour protein (gi|225432248) and putative acidic endochitinase (gi|225454408) were up-regulated. As these proteins are reported to be involved in the general response to stress (Additional file 2), their accumulation could be a secondary effect of phytoplasma infection. In contrast, ribulose bisphosphate carboxylase/oxygenase activase (gi|225449432), ribulose-1,5-bisphosphate carboxylase/oxygenase (RubisCO, gi|91984000) and chloroplastic ATP synthase CF1 alpha subunit, (gi|91983977) were down-regulated. These data suggest that leaves of recovered plants, although not showing symptoms, still have some deregulation of proteins involved in photosynthetic activity.

Most of the remaining 17 proteins, uniquely found in recovered plants, were down-regulated compared to healthy plants. Among them, we found an ascorbate peroxidase (APX), a major enzyme involved in H_2_O_2_ scavenging. Previous studies of grapevine have suggested that recovery from phytoplasma disease is associated with down-regulation of enzymatic H_2_O_2_ scavengers, supported by direct evidence of the accumulation of H_2_O_2_ in the leaves of recovered plants but not in healthy or diseased plants [15]_._ The same phenomenon has been observed in phytoplasma-recovered apple [48] and apricots [49], offering a strong support for a role for H_2_O_2_ in recovery. Our results reinforce this model: specific down-regulation of APX probably leads to a long-term accumulation of H_2_O_2_ in plant tissues which in turn contrasts with the pathogen virulence and spread within the plant host by direct antimicrobial action or secondary effects [15].

#### Phosphoproteome changes

Twelve proteins were differentially phosphorylated between recovered and healthy plants. Nine of these were also differentially phosphorylated in infected tissues: 8 displayed an increase in phosphorylation levels, while the auxin-induced protein PCNT115 isoform 1 was the only to show a decrease in phosphorylation levels in recovered tissues. The remaining proteins which were phosphorylated only in recovered plants, in comparison to healthy and FDp-infected plants, included: sedoheptulose-1,7-bisphosphatase, mitochondrial-processing peptidase and inorganic pyrophosphatase (Figure 2).

### Comparison with phosphorylated proteins in other plant species and prediction of Serine/Threonine/Tyrosine phosphorylation sites

The differentially phosphorylated proteins of *V. vinifera* were analysed by the phosphoprotein BLAST available on the P3DB database. According to the P3DB database, 15 of these proteins were already known to be targets for kinases in other plant systems, but for sedoheptulose-1,7-bisphosphatase (gi|225466690), peroxiredoxin-2B (gi|225445188) and a putative cyclase (gi|147838052), our work represents the first report of a possible phosphorylation-dependent regulation in plants (Table 1). Moreover all the 18 phosphoproteins detected by ProQ Diamond dye were also predicted to have Serine/Threonine/Tyrosine phosphorylation sites by NetPhos 2.0 software (Table 1).

**Table 1:** Bioinformatic analysis of phosphorylated proteins detected by ProQ Diamond stain

**Protein BLASTp description**	**Plant species**	**Predicted sites**		
		**Serine**	**Threonine**	**Tyrosine**
putative plastid lipid-associated protein	*Arabidopsis thaliana*	10	5	0
succinate dehydrogenase	*Oryza sativa*	15	7	1
Putative cylase, Kynurenine formamidase	**first report**	6	2	5
rubisco activase 2, chloroplastic isoform 1	*Arabidopsis thaliana*	5	3	6
soluble inorganic pyrophosphatase-like	*Arabidopsis thaliana, Madicago truncatula, Oryza sativa*	5	1	4
similar to HSC70-1 (heat shock cognate 70 kDa protein 1); ATP binding isoform 1	*Arabidopsis thaliana, Glycine max, Medicago truncatula, Oryza sativa*	15	14	5
ascorbate peroxidase	*Arabidopsis thaliana, Brassica napus, Oryza sativa*	4	3	3
putative 2,3-bisphosphoglycerate-independent phosphoglycerate mutase	*Arabidopsis thaliana, Glycine max, Oryza sativa*	12	4	12
cell division cycle protein 48 homolog	*Arabidopsis thaliana, Brassica napus, Madicago truncatula, Oryza sativa*	24	9	4
mitochondrial-processing peptidase subunit alpha	*Oryza sativa*	18	6	7
Peroxiredoxin-2B	**first report**	5	1	0
oxygen-evolving enhancer protein 2 23-kDa polypeptide of photosystem II	*Arabidopsis thaliana, Brassica napus*	8	3	4
actin-101	*Arabidopsis thaliana, Oryza sativa*	10	6	7
ribulose bisphosphate carboxylase/oxygenase activase	*Arabidopsis thaliana, Brassica napus, Madicago truncatula, Oryza sativa*	7	5	6
probable ribose-5-phosphate isomerase	*Arabidopsis thaliana*	15	3	2
auxin-induced protein PCNT115 isoform 1	*Oryza sativa*	5	5	4
ribulose 1,5-bisphosphate carboxylase/oxygenase large subunit	*Arabidopsis thaliana*	7	3	3
sedoheptulose-1,7-bisphosphatase, chloroplasic	**first report**	21	7	2

Column 1: phospho-protein list. Column 2: BLAST analysis based on P3DB database showing previous report of the same phosphorylated proteins in other plant species. Column 3: number of predicted Serine/Threonine/Tyrosine phosphorylation sites by NetPhos 2.0.

### GO functional analysis, enrichment and integration of proteome data into biological interaction networks

In order to identify possible mechanisms of regulation or functional pathways that were specifically activated in our experimental conditions, differentially expressed proteins were associated to GO terms using the plant GOslim ontology (Additional file 2). To gain deeper insight into GO distribution, an Enrichment Analysis with Hochberg FDR test was then performed. This analysis allowed us to calculate which functions were over-represented in the lists of the differentially expressed proteins identified in infected and in recovered plants with respect to healthy ones. As we were interested in the identification of the global proteomic changes in *Vitis* following FDp infection and recovery, we did not consider the quantitative and phosphorylated levels of regulation separately. This approach allowed us to rely on a large dataset to perform robust statistical analysis. Thus, we considered as differentially regulated proteins that changed both in their amount (visualized by SYPRO^®^ Ruby staining) and in their phosphorylation state (visualized by ProQ Diamond staining).

**Figure 5:**
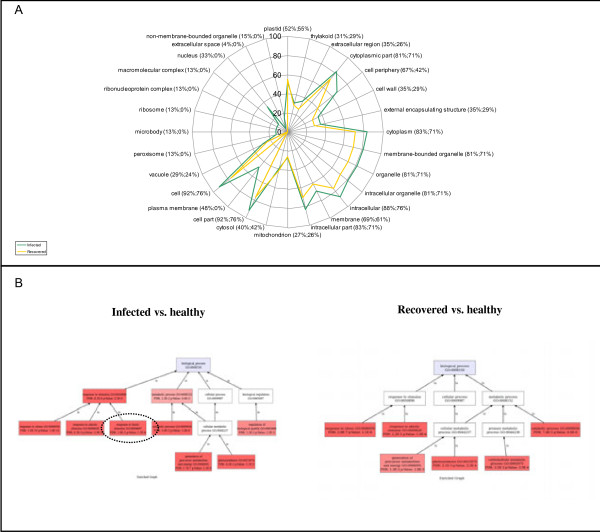
**GO classification and GO enrichment analysis of differentially expressed proteins in infected and recovered plants. A**) Cellular component term assignment and distribution. **B**) Biological process term assignment and distribution. The statistical significance of the Enrichment Analysis is represented by a scale of red tones whose intensity is proportional to the degree of significance starting from FDR ≤ 0.05.

Enrichment analysis applied to the GO cellular component terms showed that “plastid” (GO:0009536), a child category of “organelle”, was over-represented both in infected and in recovered plants (Figure 5A; Additional file 3). In fact, more than one-third of the proteins were predicted to be localized in the chloroplast (Figures 2 and 4). However, not all the chloroplast-localized proteins were related to photosynthetic reactions. For example, we found three chloroplastic enzymes involved in the response to oxidative stress, i.e. glutathione S-tranferase (gi|118406886), peroxiredoxin-2B (gi|225445188) and ascorbate peroxidase (gi|225435177), one protein that plays an essential role in the metabolism of nitrogen, i.e. glutamine synthetase (gi|147784260), and a thiamine biosynthetic enzyme (gi|225461077) (Figure 4).

The same statistical analysis was applied to the GO biological process terms (Figure 5B and Additional file 3). The results showed an over-representation of the GO categories “photosynthesis” (GO:0015979) and “generation of precursor metabolites and energy” (GO:0006091) in infected plants, supporting the perturbation of photosynthetic reactions and other metabolic ways during infection. These two categories were also over-represented in recovered plants (Figure 5B). Our data suggest that recovered plants may still show a metabolic trace of the previous phytoplasma infection, which may have caused a general perturbation of the photosynthesis. These results are likely to support the data presented by Morone and colleagues on the productivity of recovered vines, which was lower than that of healthy ones [3].

**Figure 6:**
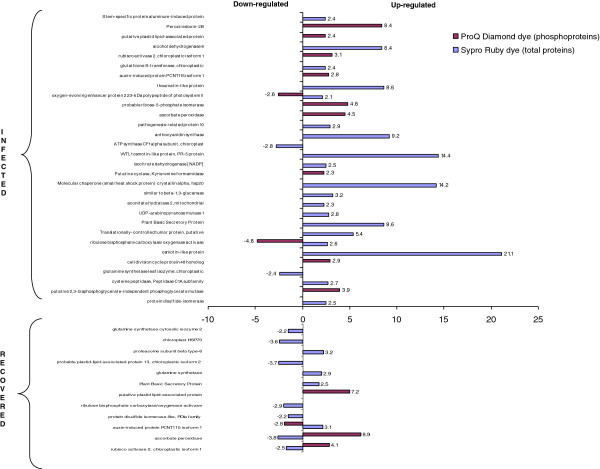
**Expression levels of proteins in the “response to stimulus” category in infected and recovered plants compared to healthy plants.** Singular Enrichment Analysis of GO categories distribution showed that the specific category “response to stimulus” (GO:0050896) was over-represented in both infected and recovered plants. The majority (71%) of the proteins belonging to this biological process was up-regulated in the infected plants, while, in contrast, 50% of the proteins was down-regulated in recovered plants compared to healthy plants. For each specific protein the fold change in expression value is reported compared to healthy plants (reference value = 1).

Regarding the stress/defence responses, the GO categories “response to stimulus” (GO:0050896) and its child categories “response to abiotic stimulus” (GO:0009628), “response to biotic stimulus” (GO:0009607) and “response to stress” (GO:0006950) were over-represented in infected plants (Figure 5B). The majority (71%) of the proteins belonging to the main category “response to stimulus” were up-regulated (Figure 6). The same category was still over-represented in recovered plants but, in contrast to what we observed in infected plants, half of the proteins belonging to this biological process was down-regulated compared to healthy plants (Figure 6). In this context, it is important to note that within the general “response to stimulus” category, the two child categories “response to abiotic stimulus” and “response to stress”, were still over-represented in recovered plants, whereas the child category “response to biotic stimulus” disappeared (Figure 5B). Overall, these results showed a general repression of proteins involved in the response to generic stress and a return to steady-state levels of those proteins specifically required for defence against pathogens.

## Conclusions

**Figure 7:**
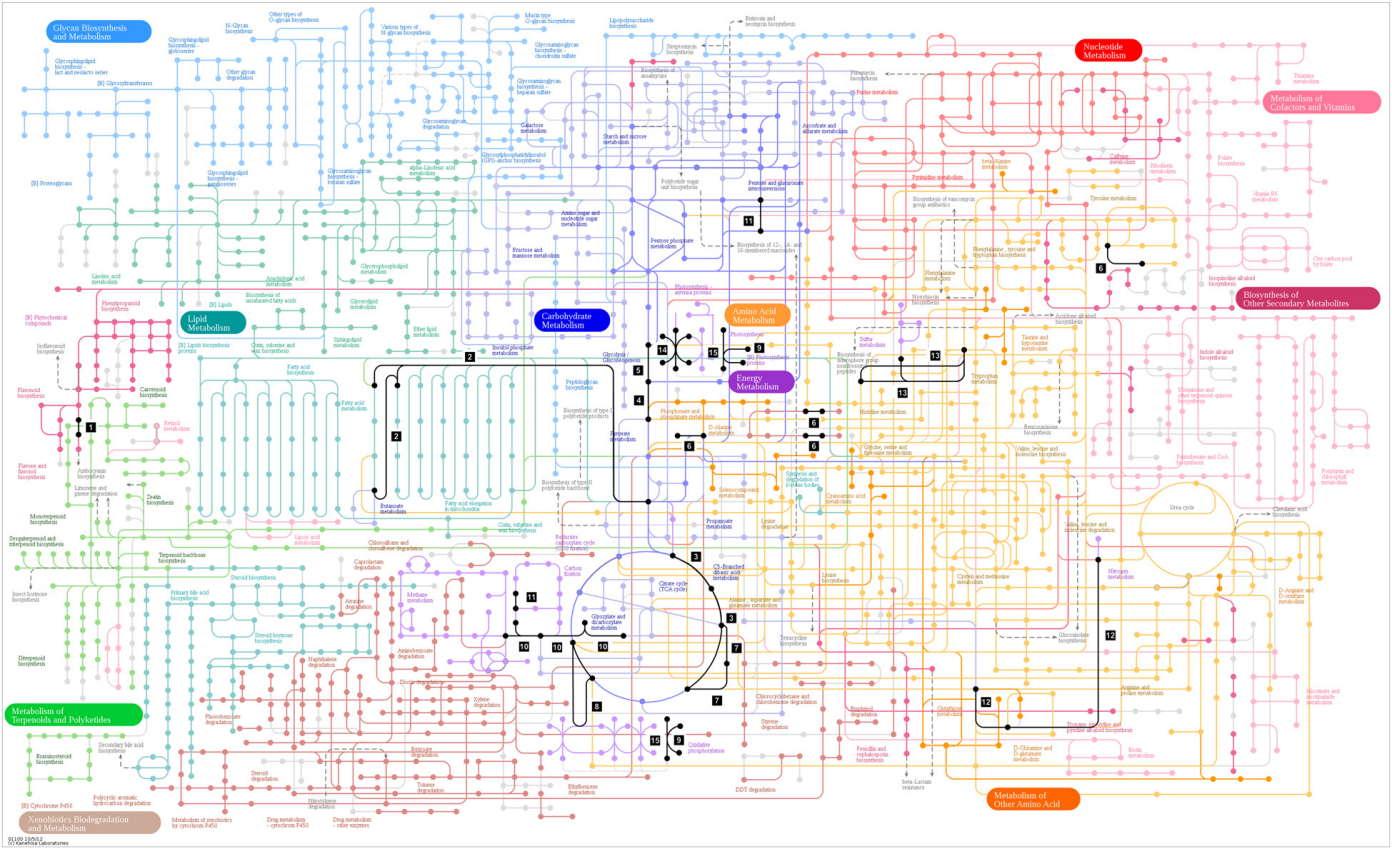
**Mapping of the differentially regulated metabolic proteins during FDp infection according to KEGG “Metabolic pathways - Reference pathway (KO)”. **The web-based server KAAS was used to display KEGG map assignments of the differentially regulated proteins. The “Metabolic pathways” map (in various colours) was the one that included the majority of the protein hits (1–15, in black). 1: anthocyanidine synthase; 2: acetyl-CoA acetyltransferase; 3: aconitate hydratase 2; 4: enolase; 5: 2,3-bisphosphoglycerate-independent phosphoglycerate mutase; 6: alcohol dehydrogenase 6; 7: isocitrate dehydrogenase; 8: succinate dehydrogenase; 9: ATP synthase CF1 alpha subunit; 10: ribulose-1,5-bisphosphate carboxylase/oxygenase large subunit; 11: ribose-5-phosphate isomerase; 12: glutamine synthetase; 13: cystathionine beta-synthase (CBS) and cysteine synthase-like protein; 14: oxygen-evolving enhancer protein 2 23-kDa polypeptide of photosystem II; 15: ATP synthase CF1 beta subunit.

In this work we have successfully used a multiplex staining approach to deeply characterize the proteome and phospho-proteome of grapevine in response to phytoplasma infection by 2-DE. Using Sybro Ruby dye, we were able to describe 37 proteins which were significantly differentially expressed between healthy and infected plants. Furthermore, by using the ProQ Diamond dye we described for the first time phosphorylation changes (15 proteins) in grapevine during pathogen infection. Based on evidences from the literature, some phosphorylated proteins were previously reported to be regulated following abiotic stress in other plants [18,38]. Our work suggests a role for their differential phosphorylation also in response to biotic stress. These findings are in good agreement with recent reports suggesting that mechanisms by which plants respond to different stresses (abiotic and biotic) are not independent, but rather crosstalk each other and share several biochemical networks at least partially overlapped [38,50-52]. Most of the proteins up-regulated in response to infection were back regulated to the steady-state level after remission of symptoms and recovery. Alterations of biological networks associated to the described proteins were predicted by bioinformatic analysis and the data were integrated to evidence inter-relationships between the different pathways (Figure 7). The data showed that phytoplasmas infection strongly affects important primary metabolisms, such as glycolysis, TCA cycle and aminoacid metabolism. These processes are fundamental for cell survival and they also provide intermediate and final products that take part in several metabolic ways. We provide a model showing the possible effects of protein modulation on these primary metabolic ways and related secondary pathways (Figure 7). In future, it will be interesting to integrate the current proteomic data with metabolic data in order to confirm the hypothesis generated by our approach. As a perspective, future approaches focused on protein characterization, functional analysis or reverse-genetic approaches will be of great support to further explore the molecular basis of grapevine susceptibility to phytoplasma infection and recovery.

## Methods

### Plant material collection

Field-grown grapevine samples were collected in Cocconato, Asti province (Piedmont, north-western Italy), in summer (August) 2009. The vineyard had been monitored since 2005 for phytoplasma infection and a map of the sanitary status of the plants (healthy, infected and recovered) was available at the beginning of this study. All samples were collected based on symptom observation and previous monitoring data: healthy plants did not show any suspicious symptoms, whereas infected plants showed at least three typical symptoms of phytoplasma infection [53]. Recovered plants had tested positive for FDp infection (also showing strong specific symptoms) at least one year before material collection for our proteomic analysis, and did not show symptoms or test positive for phytoplasmas in the following years. In order to minimize the potential variability, which is un-avoidable when working in field conditions, all the plants studied were grapevines propagated from the same initial clone and collected in the same day. Moreover, to normalize the multiple parameters in field conditions (for example differences in light, soil composition and drainage), the selected plants were collected nearby each other. The plants were regularly treated with fungicides and no typical symptoms of fungal disease were observed during sampling. All samples were tested for phytoplasma infection using molecular assays [54], and any mixed infections with Bois noir phytoplasma were excluded from further work. In addition, we tested the samples for the presence of the most common viruses reported in Piedmont vineyards, some of which can show similar symptoms to phytoplasmas. According to methods in the literature [55], we tested the following eight viruses using RT-PCR assays: Grapevine Virus A (GVA), Grapevine Virus B (GVB), Grapevine Fanleaf Virus (GFLV), GFkV, Grapevine Leaf-Roll-Associated Viruses (GLRaV) 1, 2 and 3 and Arabis mosaic virus (ArMV). Three FDp-negative plants, three FDp-positive plants and three recovered plants were chosen as the biological replicates in the 2-DE experiments. Leaf midribs were cut with a scalpel and pooled together to prepare 0.5 g samples for RNA extraction and 1 g samples for total protein extraction. The material was frozen in liquid nitrogen and stored at −80°C until further use.

### Protein extraction and 2-DE

Total proteins were extracted using a TCA/acetone method [7] using proteinase and phosphatase inhibitor cocktails (Roche Diagnostics, Mannheim, Germany). The final pellet was resuspended in rehydration solution (8 M urea, 2% (w/v) 3-[(3-cholamidopropyl)dimethylammonio]-l-propanesulphonate idrate (CHAPS), 50 Mm Dithiothreitol (DTT), 0.2% (v/v) Bio-Lyte 3/10 BioRad ampholites), and total protein concentration was assessed using the ‘2-D Quant Kit’ (GE Biosciences, Uppsala, Sweden), using Bovine Serum Albumine (BSA) as standard. Isoelectrofocusing was carried out using 7 cm-long ReadyStrips IPG Strips, pH interval 4-7 (BioRad, Hercules, CA, USA), as preliminary assays showed high concentrations of total and phosphoproteins within this pH range. The running conditions for the first dimension were: passive rehydration for 14 h at 20°C, followed by a linear voltage ramping until 4000 V was reached and focusing on a 4000 V constant voltage until 14000 V h, in a Protean IEF Cell (BioRad), with a maximum current of 50 μA/strip. Strips were stored at −80°C until the second dimension electrophoresis was to be performed. For the second dimension, the strips were equilibrated twice upon gentle agitation for 20 min in an equilibration buffer (6 M urea, 2% (w/v) sodium dodecyl sulphate (SDS), 375 mM Tris–HCl pH 8.8, 20% (v/v) glycerol), containing 60 mM DTT the first time (reduction step) and 2.5% (w/v) iodoacetamide the second time (alkylation step). Poly-acrylamide gel electrophoresis was performed by placing the focused strips on vertical 12% poly-acrylamide gels, according to the Laemmli buffer (25 mM Tris, 192 mM glycine, 0.1% SDS) system, in Protean II cell (BioRad), at 150 constant volts, until the blue front reached the bottom of the gel. Three replicate gels were run for each biological sample. The Precision Plus Protein Standards-All Blue (BioRad) and PeppermintStick phosphoprotein molecular weight standards (Invitrogen, Carlsbad, CA, USA) were used as MW marker and positive/negative control for phosphorylation staining.

### Multiplex staining and image analysis

After 2 -DE, the gels were treated with fixing buffer (50% (v/v) methanol, 10% (v/v) acetic acid) and then washed three times in water for 10 min. To detect phosphoproteins, the gels were stained using the Pro-Q Diamond phosphoprotein gel stain kit (Invitrogen). The manufacturer’s protocol was followed, except that half (50 ml) of the recommended quantity of stain was used, in order to reduce the background, as shown by our preliminary work and other authors [17]. Gels were then destained by incubation (30 min, two times) in 100 ml of destaining solution (20% acetonitrile, 20 mM sodium acetate, pH 4.0) and washed in water (5 min, two times). After fluorescence scanning (Versadoc Imaging System, BioRad), the gels were washed three times with water and incubated with 50 ml of Sypro^®^Ruby stain (Invitrogen). The staining, destaining and washing procedures were performed as suggested by the manufacturer. The gel images were analysed with PDQuest version 8.0.1 software (BioRad). Manual inspection of the spots was performed to verify the accuracy of automated gel matching and any errors or missing spots in the automatic procedure were manually corrected prior to final data analysis. Gel image analysis was first conducted separately on ProQ Diamond and Sypro^®^Ruby stained gels. Normalization was set up according to the total spot density. Only spots that could be detected in all the biological and technical replicates were considered for further analysis.

Total proteome analysis of SyproRuby-stained gels was performed by creating a statistical (ANOVA test, p < 0.05) and a quantitative (volume variation by at least a factor of two) analysis set. Next, a Boolean analysis set that identified the spots common to the previous sets was created to select the differentially expressed spots. Phosphoproteome analysis was performed according to published methods [56,57]. Spots common to both ProQ Diamond and Sypro^®^Ruby- stained gels were selected and their values compared by software to normalize the phosphorylation value to the expression value for each matched spot. Spots with p < 0.05 according to ANOVA and | ratio | > 2 were considered differentially phosphorylated and selected for further analysis. The experimental molecular weights and iso-electric points of the spots were derived according to the standards.

### Mass spectrometry

Mass spectrometry was performed at the Proteomics Technology Facility, Department of Biology, University of York (http://www.york.ac.uk/depts/biol/tf/proteomics). Protein spots were removed from preparative gels derived from 17-cm long strips, stained with Coomassie Brilliant Blue, destained, trypsin digested and analysed by mass spectrometry (Ultraflex III MALDI TOF/TOF mass spectrometer, Bruker, Billerica, MA, USA) according to the method previously described [7].

The FlexAnalysis software version 3.3 (Bruker) was used to perform the spectral processing and peak list generation. The mass spectra were analysed using the MASCOT peptide sequence matching software (Matrix Science Ltd., version 2.1) through a Bruker ProteinScape interface (vs. 2.1), against the NCBI *Vitis Vinifera* database and the unrestricted NCBI database. The last search was performed in order to a) extend the number of searchable proteins and possibly find significant assignments to proteins with no matches in the *Vitis* database, b) find possible contaminants. The search parameters were as follows: Enzyme, Trypsin; Fixed modifications, Carbamidomethyl; Variable modifications, Oxidation; Peptide tolerance, 250 ppm; MS/MS tolerance, 0.5 Da; Max missed cleavages, 1. The significance threshold was inferred at p < 0.05. The theoretical mass and p*I* of the proteins identified were calculated from sequence data with the Expasy Compute p*I*/Mw tool (http://www.expasy.ch/tools/pi_tool.html).

Only peptide mass maps that matched single annotated *Vitis* proteins were kept in the final database (Figure 2, Additional file 1), whereas those matching only bacterial or human proteins were excluded. Although the majority of spots identified corresponded to a single protein, there were a few spots where MALDI TOF/TOF data indicated a mixture of multiple protein species, which was likely due to proteins co-migrating in one spot. These proteins were excluded from the final database. It never occurred that a peptide with no matches in the *Vitis* database found a match with any other plant protein in the extended search.

### Bioinformatic analysis

The *V. vinifera* annotation file was downloaded from B2G-FAR website (http://www.b2gfar.org) and loaded into Blast2GO [58]. The already existing annotation was further integrated by a Plant GOslim classification of the Vitis proteome. Then, the whole dataset was used as “reference set” to determine the over-representation of Plant GOslim terms and their corresponding P-values by Enrichment Analysis (Fisher’s Exact Test) in infected and in recovered plants (used as “test sets”), separately. The Hochberg FDR-controlling method was chosen as a multiple testing correction. Those Plant GOslim categories with a P-value less than 0.05 after the FDR correction were considered significantly over-represented.

The web-based functional annotator KAAS (KEGG Automatic Annotation Server) was used to assign the differentially regulated proteins to KEGG pathways.

To identify newly reported phosphorylated proteins, differentially phosphorylated proteins were analysed by the Phosphoprotein BLAST available on the P3DB database (http://www.p3db.org) and compared to protein phosphorylation data for six plant species (*A. thaliana*, *Brassica napus*, *Glycine max*, *Medicago truncatula*, *Oryza sativa* and *Zea mays*).

The presence of potential Serine/Threonine/Tyrosine phosphorylation sites was predicted by NetPhos 2.0 software (http://www.cbs.dtu.dk/services/NetPhos).

## Competing interests

The authors declare that they have no competing interests.

## Authors’ contribution

PM participated in the design of the study, collected samples, carried out the proteomic analysis, participated in the bioinformatic analysis, and wrote the manuscript; SA performed the bioinformatic analysis and helped with manuscript writing; SP conceived the study and its design, coordinated the activities, and drafted and edited the manuscript. All authors read and approved the final manuscript.

## Supplementary Material

Additional file 1 **Detailed list of the differentially expressed proteins.** The following parameters are reported for each spot: sequence of the matched peptides, normalized volume values (PPM) and coefficient of variation, statistical significance. Sheet 1: healthy/infected Sypro^®^ Ruby staining (total proteins); sheet 2: healthy/recovered Sypro^®^ Ruby staining; sheet 3: healthy-infected ProQ Diamond staining (phosphoproteins); sheet 4: healthy-recovered ProQ Diamond staining. Click here for file

Additional file 2 **GO classification and KEGG map assignments of the identified *V. vinifera *proteins. **GOslim term assignment was performed by Blast2GO. GO classification is expressed by GO Names (third column) preceded by “P” for “biological process”, “C” for cellular component and “F” for “molecular function”. The forth column of the table reports the names of the KEGG maps assigned to each protein according to the KAAS functional annotation. Click here for file

Additional file 3 **List of the over-represented GO categories in infected and recovered plants following Singular Enrichment Analysis. **Proteins are grouped according to the GO categories to which they belong. Only the statistically significant (False discovery rate: FDR ≤0.05) GO terms are shown. “Category” indicates the main GO categories: “B”, biological process; “C”, cellular component. There were no statistically significant molecular function terms. “Number of sequences” indicates the number of proteins assigned to each GO term. Click here for file
